# Independent components of human brain morphology

**DOI:** 10.1016/j.neuroimage.2020.117546

**Published:** 2021-02-01

**Authors:** Yujiang Wang, Karoline Leiberg, Tobias Ludwig, Bethany Little, Joe H Necus, Gavin Winston, Sjoerd B Vos, Jane de Tisi, John S Duncan, Peter N Taylor, Bruno Mota

**Affiliations:** a CNNP Lab (www.cnnp-lab.com), Interdisciplinary Complex Systems Group, School of Computing, Newcastle University, Newcastle upon Tyne, UK; b Faculty of Medical Sciences, Newcastle University, Newcastle upon Tyne, UK; c UCL Queen Square Institute of Neurology, London, UK; d Graduate Training Center of Neuroscience, University of Tübingen, Tübingen, Germany; e Centre for Medical Image Computing (CMIC), University College London, London, UK; f Department of Medicine, Division of Neurology, Queen’s University, Kingston, Canada; g Epilepsy Society MRI Unit, Chalfont St Peter, UK; h Institute of Physics, Federal University of Rio de Janeiro, Brazil

## Abstract

Quantification of brain morphology has become an important cornerstone in understanding brain structure. Measures of cortical morphology such as thickness and surface area are frequently used to compare groups of subjects or characterise longitudinal changes. However, such measures are often treated as independent from each other. A recently described scaling law, derived from a statistical physics model of cortical folding, demonstrates that there is a tight covariance between three commonly used cortical morphology measures: cortical thickness, total surface area, and exposed surface area. We show that assuming the independence of cortical morphology measures can hide features and potentially lead to misinterpretations. Using the scaling law, we account for the covariance between cortical morphology measures and derive novel independent measures of cortical morphology. By applying these new measures, we show that new information can be gained; in our example we show that distinct morphological alterations underlie healthy ageing compared to temporal lobe epilepsy, even on the coarse level of a whole hemisphere. We thus provide a conceptual framework for characterising cortical morphology in a statistically valid and interpretable manner, based on theoretical reasoning about the shape of the cortex.

## 1 Introduction

Since magnetic resonance imaging has become widely available, the quantification of brain morphology has become a standard tool. Differences in brain morphology between a control and a comparator cohort are often reported for many processes in health and disease. Alterations in brain morphology, however, may be non-specific; many processes appear to be associated with similar changes. For example, in healthy ageing, many studies report a thinning of the cortex as the predominant characteristic (e.g. Bajaj, Alkozei, Dailey, Killgore, 2017, Hutton, Draganski, Ashburner, Weiskopf, 2009). Similarly, many brain disorders (e.g. bipolar disorder Hibar et al., 2018, schizophrenia van Erp et al., 2018, temporal lobe epilepsy Whelan et al., 2018, and Alzheimer’s disease Dickerson et al.) also feature cortical thinning as the predominant cortical alteration compared to controls. Such observations can lead to naïve conceptualisations, e.g. that the biological processes determining cortical thickness are particularly “fragile”, or that certain brain disorders are the result of “premature ageing”. In this study, we demonstrate that such concepts are inferences based on a univariate view of the brain morphology data. When considering a multivariate view, accounting for covariance, the alterations in different processes can be shown to be more specific and distinct.

One such multivariate view of brain morphology data has been proposed in the context of quantifying cortical folding. Based on a statistical physics model describing cortical folding, Mota *et al.* predicts that cortical thickness T, cortical (pial) surface area $A_{t},$ and exposed surface area $A_{e}$ should be tightly linked by a scaling law $A_{t}\sqrt{T}=kA_{e}^{5/4},$ where k is a constant. The exposed surface area is often described as the area of the cortex that is visible on the outside. This equation has been derived based on the assumption that the cortex is a tissue of finite thickness that folds in a way that balances compressive mechanical forces with the imperative that it must be self-avoiding. The resulting scaling law has been confirmed by empirical data across mammalian species (Mota and Herculano-Houzel, 2015), individual humans (Wang et al., 2016), and even across different lobes of the same brain (Wang et al., 2019b). This scaling implies a tight covariance of the three morphological variables, whereby changes in one variable must be balanced by changes in the other variables. Conceptually, this means that, for example, if cortical thickness and total surface area are specified (by, e.g., the specifics of various neuroproliferative pathways during development), then its exposed area and volume follow as a physical consequence. More succinctly, cortical morphology variables are not independent of each other and cannot vary freely.

The practical implication of the scaling law is that the three morphological quantities of cortical thickness, cortical surface area, and corresponding exposed surface area should not be treated independently when assessing brain morphology. Independent comparisons of these quantities may result in incorrect conclusions when not accounting for the covarying morphological features. For example, comparing cortical thickness between two groups without accounting for differences in surface area and exposed area (morphological covariates) would be as naive as comparing an Alzheimer’s group against a control group without accounting for group differences in age (a biological covariate).

Is there then a more systematic way of analysing cortical morphology that accounts for the covariance between morphological variables? The scaling law itself provides a natural way forward. In mathematical terms, the scaling law provides the first component of a “principal component” decomposition of the three morphological variables. We show how to generate two further well-motivated components, so that all three are independent of each other and can be used to specify cortical morphology. We will demonstrate this principle and show that the effects of brain disorders (temporal lobe epilepsy in our example) that *appear* morphologically similar to ageing in naive univariate analyses are actually distinct, if the full set of new independent components are taken into account.

## 2 Methods

### 2.1 Data and demographics

To study the alterations associated with ageing, we used T1 and T2 weighted MRI brain scans from the Cambridge Centre for Ageing and Neuroscience (Cam-CAN) dataset (available at http://www.mrc-cbu.cam.ac.uk/datasets/camcan/ Shafto et al. (2014); Taylor et al. (2017)). Cam-CAN used a 3T Siemens TIM Trio System with 1 mm isotropic voxel size (for more details see Shafto, Tyler, Dixon, Taylor, Rowe, Cusack, Calder, Marslen-Wilson, Duncan, Dalgleish, et al., 2014, Taylor, Williams, Cusack, Auer, Shafto, Dixon, Tyler, Henson, et al., 2017). From the Cam-CAN dataset we retained 644 subjects that successfully completed preprocessing (recon-all – see next section) without errors. From these subjects we selected all subjects between 23 and 27 years old (inclusive) as our reference cohort, and all subjects between 33 and 37 (inclusive) as the comparison cohort. This resulted in 34 subjects in the reference cohort and 56 subjects in the comparison cohort. Note that in Supplementary Data, we show results for more groups from the Cam-CAN dataset to demonstrate robustness of the results.

To study the alterations associated with temporal lobe epilepsy (TLE), we used the same subjects (patients and controls) as in Taylor et al. (2018) and focused on the T1 weighted images. This dataset was obtained on a 3T GE Signa HDx scanner (General Electric, Waukesha, Milwaukee, WI) using a coronal T1-weighted volumetric acquisition with 170 contiguous 1.1 mm thick slices (matrix, 256×256; in-plane resolution, 0.9375×0.9375 mm), for more details see Taylor et al. (2018). The TLE dataset included 53 patients with TLE (comparison cohort) and 30 controls (reference cohort). The control cohort spans an age range of 19-64 years, and the TLE cohort spans an age range of 19-67 years. Note that the TLE dataset is never directly compared to the CamCAN, we only perform comparisons within datasets and obtain effect sizes within datasets.

### 2.2 Data processing

The MR images of both datasets were first preprocessed by the FreeSurfer 6.0 pipeline *recon-all*, which extracts the grey-white matter boundary as well as the pial surface. These boundaries were then quality checked and manually corrected where needed. Next, the relevant quantities (pial surface area, cortical thickness, and exposed surface area) were extracted from the FreeSurfer output files and assembled into one table (code is available in Wang and Ludwig (2019)). Note the exposed surface area is obtained as part of the LGI pipeline (Schaer et al., 2008) in FreeSurfer (?h.pial-outer-smoothed). In the following, the analysis is always hemisphere based, as in our previous work (Mota, Herculano-Houzel, 2015, Wang, Necus, Kaiser, Mota, 2016). We did not perform a more regionalised analysis in the main results, which is also possible (Wang et al., 2019b), as we wish to demonstrate the principle of independent morphological variables rather than describe the exact nature of morphological changes in a particular process. Future work using the principle demonstrated here can be directly extended to include regionalised measures, as we show in Suppl. Text S3 and discuss later.

### 2.3 Scaling law analysis, and new morphological measures

Throughout the paper, we use a log-space representation of all variables to allow expressing products of power laws as linear combinations. We also chose variables that have all dimensions of area ($A_{t},A_{e}$ and $T^{2}$), to allow an easier interpretation of the combination of variables. In this representation, each cortex corresponds to a point in three-dimensional space with coordinates $\vec{p}=\left\{A_{t},A_{e},T^{2}\right\},$ and the scaling law $\log A_{t}+\frac{1}{4}\log T^{2}=\log k+\frac{5}{4}\log A_{e}$ defines the plane close to which most cortices are situated. By isolating the parameter k, we obtain $K=\log k=\log A_{t}-\frac{5}{4}\log A_{e}+\frac{1}{4}\log T^{2},$ which is the projection of $\vec{p}$ along $\vec{\kappa }=\left\{1,-\frac{5}{4},\frac{1}{4}\right\}$. In short, $K=\vec{p}\cdot \vec{\kappa }$. We have previously hypothesised that the near invariance of K is a tension/pressure that is applied to the cortical tissue (Mota, Herculano-Houzel, 2015, Wang, Necus, Kaiser, Mota, 2016). We thus call K the tension term. Note that K is almost constant for a homogeneous adult cohort of human subjects (Wang et al., 2016), and varies little across species (Mota and Herculano-Houzel, 2015).

Remarkably, K is a dimensionless quantity. This means that if two cortices are isometrically scaled versions of one another (i.e., same shape, different size), they will have the same K value. Mathematically, isometric scaling means all areas, $A_{t},$ $A_{e},$ and $T^{2},$ are multiplied by a common numerical factor. This corresponds to movement perpendicular to $\vec{\kappa }$ in the direction $\vec{\iota }=\left\{1,1,1\right\},$ the projection to which yields the so-called isometric term $I=\vec{p}\cdot \vec{\iota }$. For a third and last element of our new set of orthogonal vectors, we use the cross-product of $\vec{\kappa }\times \vec{\iota }=\vec{\sigma }=\left\{\frac{3}{2},\frac{3}{4},-\frac{9}{4}\right\},$ the direction that is perpendicular to both $\vec{\kappa }$ and $\vec{\iota }$. The resulting projection $S=\vec{p}\cdot \vec{\sigma }$ is the corresponding shape factor.

The scalar value I captures all the information about the size of the structure. Changing I, while keeping the other parameters constant, corresponds to isometrically shrinking or expanding a shape. One can think of the term I, calculated for any particular shape, as a measure of size that carries no information about shape.

Conversely, the plane $\vec{\kappa }\times \vec{\sigma },$ henceforth called the isometric plane, carries only information about shape, and is not affected by size or changes in overall scaling. Any direction in this plane corresponds to the logarithm of a dimensionless parameter (mathematically, the sum of its vector coefficients is zero).

In our definition of the new components, $\vec{\kappa },$ $\vec{\sigma }$ and $\vec{\iota }$ have different length (as opposed to having unit length). This is not problematic in our analysis, as we standardise (z-score) all subjects relative to the reference group in log space of K, I, and S. However, future application may want to use normalised vectors.

### 2.4 Age and sex correction

In order to investigate the effect of temporal lobe epilepsy alone, without the confounding effects of age and sex, we linearly regress out the effect of age and sex from all three log-transformed morphological variables cortical thickness, cortical surface area, and exposed surface area. We do this by deriving the linear regression coefficients from the control cohort, and applying them to both the control and the patient cohorts. Interaction between age and sex was not modelled.

To study the effect of ageing, we used two groups within a small age range (23–27 years old *vs.* 33–37 years old). Thus, we did not perform the age correction, but only a sex correction by performing a mean centering for both sexes independently.

### 2.5 Statistical analysis

To statistically compare the effects of ageing and temporal lobe epilepsy, we standardise all quantities relative to the respective control cohort and report all effects in terms of effect sizes. This was achieved by converting all measures in all subjects to z-scores relative to the mean and standard deviation of the respective reference/control cohort. Hence, all quantities reported are in terms of z-scores. To measure the mean difference between the reference cohort and the comparison cohort (older ageing group or TLE cohort), we show the distribution of bootstrapped means (over 100 resampling iterations) of the z-scores for both groups as violin plots. Note that as we are using a distribution of bootstrapped means, the mean of this distribution should be very close to zero in the reference groups, but may not be exactly zero in all cases due to the stochastic nature of bootstrapping.

To measure average effect between groups (termed d in the following), we then form the difference between the average bootstrapped means (of reference *vs.* comparison groups). Note that d is positive if the comparison group (older age group, or TLE) has a higher mean value than the reference group, and *vice versa*.

As we were interested in group effects of ageing and TLE, we focused our attention on the group mean estimation. The bootstrapping was applied as a data-driven method to obtain a more representative mean group effect that was not driven by few outliers.

We also report p-values for statistical significance in the comparison of groups, only with the purpose to be consistent with previous studies, but not for subsequent use (e.g. to select features). All *p*-values are calculated using the Wilcoxon ranksum test on the raw data (i.e. not the bootstrapped means).

### 2.6 Data availability

Code for extraction of raw cortical measures can be found on Zenodo Wang et al. (2019a) and Github: https://github.com/cnnp-lab/CorticalFoldingAnalysisTools.

Data underlying the figures in this paper and the corresponding code can be found on Github: https://github.com/cnnp-lab/2020Wang_TLEFoldingHemi

## 3 Results

### 3.1 Morphological changes in TLE appear to be the same as in ageing

**Fig. 1:**
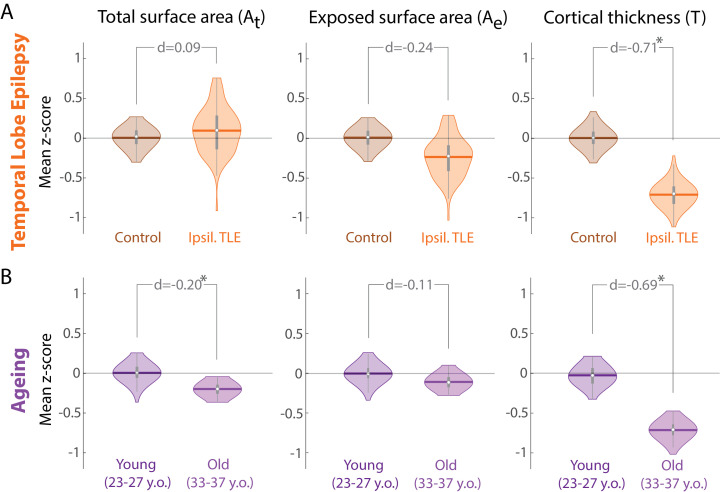
**Morphology changes in TLE appear similar to those in healthy ageing. (A)** Morphology changes in TLE in the ipsilateral hemisphere compared to a control cohort measured as z-scores. Violin plots show the distribution of bootstrapped mean z-scores. Age and sex correction was performed before the comparison. **(B)** Morphology changes in healthy ageing comparing a younger and older group of adult subjects, measured as z-scores relative to the younger subject group. Violin plots show the distribution of bootstrapped mean z-scores. Sex correction was performed before the comparison. (A & B) All morphological measures are in terms of a whole cortical hemisphere and log-scaled before analysis. Each hemisphere was treated as a separate datapoint. * denotes statistical significance at p<0.05. Beeswarm plots with raw data points are presented in Supplementary Data.

In many diseases, average cortical thickness is the most consistently decreasing variable relative to controls. Temporal lobe epilepsy (TLE) is no exception. In our data (Fig. 1), average cortical thickness of the entire ipsilateral hemisphere (cortical ribbon) is substantially reduced in patients relative to controls (d=−0.71, p=0.0008). Total and exposed surface areas do not appear substantially altered (|d|<0.3, p>0.05).

The same patterns of alteration are observed in the healthy ageing process. In our cross-sectional data, cortical thickness is substantially reduced in older subjects (d=−0.69, p=0.00004), while surface areas remain relatively unaltered.

Given these parallel alterations in brain morphology, a non-critical analysis might liken a disease conditions such as TLE to the ageing process in terms of whole-brain morphology. However, we will demonstrate in the following section that this would be an erroneous conclusion based on raw and, in this case, less informative measures of brain morphology, analysed in a univariate manner, neglecting the covariance between these measures.

### 3.2 The universal scaling law describes covariance of raw morphology measures

**Fig. 2:**
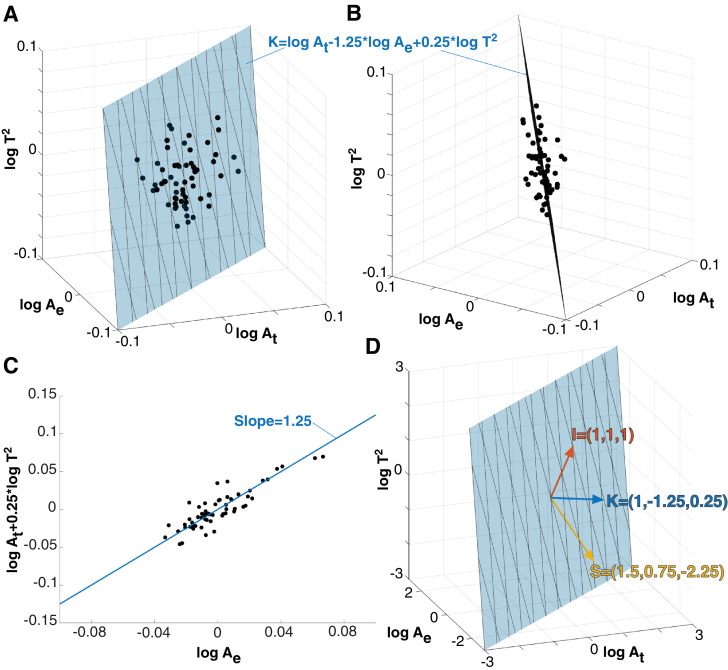
**Universal scaling law describes the covariance of the raw morphological measures. (A)** Three raw morphoplogy measures span a 3D space, where each cortex is a data point (black dots). Here we used the control group in the TLE dataset as an example for the purpose of illustration. The data points align with the plane described by the universal scaling law (blue plane). **(B)** different viewing angle of the same data shown in (A). **(C)** Projection of data into a 2D space, which was previously used to visualise the scaling law. The blue line now represents the projected plane from (A) and (B). **(D)** 3D view of scaling law plane and viewing angle as in (A). The normal vector of the scaling law plane (K) is shown as a blue vector. Two perpendicular vectors (S and I) can be defined, and together they span the 3D space. All morphological variables are logscaled and age corrected in this figure. Cortical thickness is presented as thickness squared so that the 3D space has units of area in all dimensions.

Any given cortex can be represented as a point in the $\log A_{t}\times \log A_{e}\times \log T^{2}$ space (Fig. 2A,B), which has units of area in all dimensions. By plotting the TLE control cohort in this way, it is evident that the raw morphological measures $A_{t},$ $A_{e},$ and T covary tightly in this space (Fig. 2). When superimposing the plane described by the scaling law ($\log A_{t}+\frac{1}{4}\log T^{2}=\alpha \log A_{e}+\log k,$ where α is theoretically predicted to be $\frac{5}{4}$), we can see that it fits well to describe the covariance of the raw morphological measures (Fig. 2A,B,C). Both the TLE control, as well as the patient group follow this scaling law (α slope 95% CI 1.1548 - 1.4260 and 0.9665 - 1.2827, respectively). Note that because of age-correction, all controls align on the plane described by logk=K=0.

In other words, the scaling law provides a decomposition of the raw morphological measures: The normal vector to the plane is {1,−1.25,0.25} (Fig. 2D) where the first, second, and third dimensions are $A_{t},A_{e}$ and $T^{2},$ respectively. By calculating $K=\log A_{t}-1.25\log A_{e}+0.25\log T^{2}$ we can obtain a value for K for every cortex from their raw morphological measures $A_{e},A_{t}$ and T. Based on our model (Mota and Herculano-Houzel, 2015), K can be interpreted as tension/pressure applied to the cortical tissue (Wang, Necus, Kaiser, Mota, 2016, Wang, Necus, Rodriguez, Taylor, Mota, 2019), we thus call K the tension term.

Change along the vector {1,1,1}, corresponding to isometric scaling (i.e. changing $A_{t},$ $A_{e}$ and $T^{2}$ by the same proportion, thus stretching/shrinking the brain in all direction equally) is perpendicular to the previous normal vector. We choose this to be the second component as it has a direct interpretation, and it is also independent of K in our dataset (Pearson’s ρ=0.09, p=0.45 across the TLE controls). Again, it can be calculated as $I=\log A_{t}+\log A_{e}+\log T^{2}$ (isometric term) from the raw morphological variables. It can be understood to carry information about the size of the cortex only, without containing any information about shape. Indeed, we found I to be highly correlated (Pearson’s ρ≥0.9) with several metrics of brain volume, particularly grey matter volume.

The third perpendicular vector is the cross-product of the two previous ones is $\left\{\frac{3}{2},\frac{3}{4},-\frac{9}{4}\right\}$. We will call this the shape term, and again we can calculate it as $S=\frac{3}{2}\log A_{t}+\frac{3}{4}\log A_{e}-\frac{9}{4}\log T^{2}$. Again, K and S are independent (Pearson’s ρ=0.02, p=0.85 across the TLE controls). While I only carries information about size, K and S only carry information about shape. This also means that for the same K (which is the case for all healthy human adults of the same age (Wang et al., 2016)), S is the only term that describes any changes in shape.

Here, the choice of I and S did not follow a data-driven principal component analysis. Instead, we choose directions predicted by the scaling law and that can be interpreted in biological terms, as our intention is to provide an illustrative demonstration of a set of new independent morphological variables. For completeness, we present results using a standard PCA in Supplementary Text 1. We note that PCA found a similar principal direction to K with a small (30 degree) difference. We further observed similar results upon analysing the data projected onto this PC (TLE and ageing processes differ in their direction of change), albeit with much smaller effect sizes (d=0.15 in TLE and d=-0.15 in ageing). Results in projections onto other PCs do not differ between TLE and ageing.

### 3.3 The universal scaling law defines a new set of independent morphological measures

**Fig. 3:**
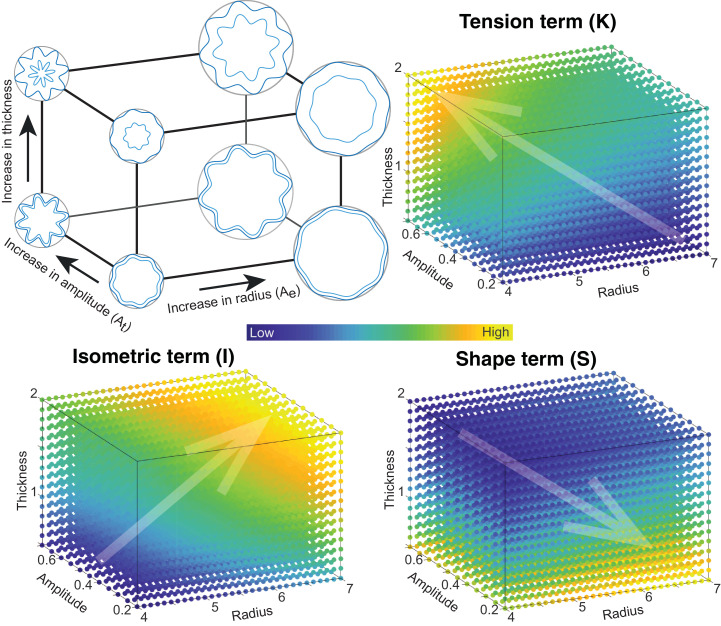
**Schematic to provide intuition for the three projection terms K, S, and I** Simulations of basic folded ribbons as sinusoidal oscillations on a circle. In this shape we can change the overall radius of the encapsulating circle ($A_{e}$), the thickness (T) encapsulated by the outer and inner oscillations (dark and light blue), and the amplitude of the oscillations, which dictate the total length of the oscillation ($A_{t}$). By scanning the radius, thickness, and oscillation amplitude in a 3D space, we can calculate the corresponding value for the K,S, and I term at different points in this space (colour map). Transparent arrows point in the directions of change of K,S, and I. Through visualising the changes in K,S, and I in this 3D space, we provide an intuition for how the three terms relate to parameters in a simple folded structure.

To provide an intuitive understanding of the new morphological variables, we provide a schematic illustration of the variables on a 2D shape (a circular sinusoidal ribbon with 8 folds) in Fig. 3. Note that this is not a mechanistic simulation of how the brain folds, but rather only intended to provide a visual way of understanding the new coordinate system K,I and S. We can intuitively parametrise this circular sinusoidal ribbon with the overall radius of the circle, the amplitude of the sinusoid, and the thickness of the ribbon to describe changes in $A_{e},A_{t}$ and T, respectively. We then demonstrate how such changes map onto the new morphological variables K,S and I. I corresponds to a measure of size, as expected. It increases with the thickness of the ribbon as well as the overall circle radius, for a constant sinusoid amplitude. S increases with a combination of overall radius and amplitude of the sinusoid, but decreases with thickness of the ribbon. Finally, K increases with thickness and sinusoid amplitude, but decreases with overall radius.

### 3.4 The scaling law derived morphological measures show differences between TLE and ageing

**Fig. 4:**
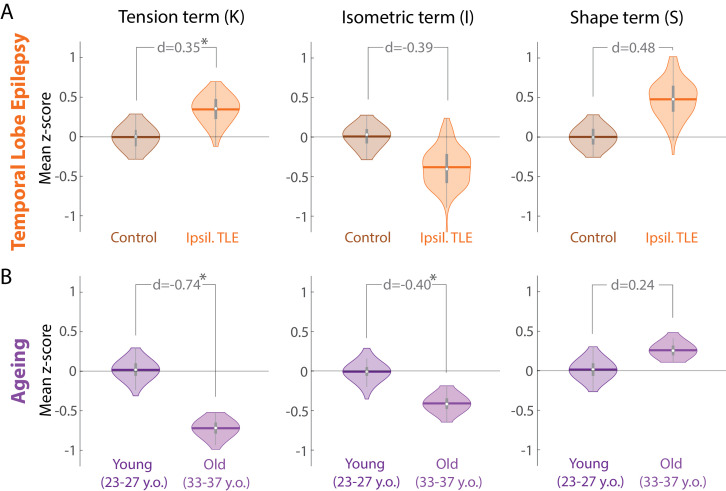
**Morphological changes in K differ in TLE compared to healthy ageing. (A)** Morphological changes in K,S, and I in the ipsilateral hemisphere in TLE compared to a control cohort measured as z-scores relative to controls. Violin plots show the distribution of bootstrapped mean z-scores. Age and sex correction of original morphological measures was performed before the comparison. **(B)** Morphological changes in healthy ageing comparing a younger and older group of adult subjects, measured as z-scores relative to the younger subject group. Violin plots show the distribution of bootstrapped mean z-scores. Sex correction of original morphological measures was performed before the comparison. (A & B) All morphological measures are in terms of a whole cortical hemisphere. Each hemisphere was treated as a separate datapoint. * denotes statistical significance at p<0.05. Beeswarm plots with raw data points are presented in Supplementary Data.

Equipped with the new measures K, S, and I, we now re-examine our initial observation that the morphological alterations in TLE resemble those of ageing. Fig. 4 shows that both ageing, and TLE are associated with similar alterations in S and I with a similar effect size (decrease in I with d≈−0.4, and an increase in S with d=0.24 and d=0.48). However, TLE is associated with an increase in K compared to controls (d=0.35), whereas ageing is clearly associated with a strong decrease in K (d=−0.74). In other words, in terms of the tension term, brain morphological differences in TLE differ from changes that occur during healthy ageing.

### 3.5 Outlook: trajectories of disease and ageing processes

We can additionally visualise the average effects from Fig. 4 as datapoints in the three-dimensional space of spanned by K×S×I, in terms of effect sizes in each of those three independent variables. In other words, each process/condition (ageing, TLE) can be understood as an alteration in K, S, and I relative to controls/reference. By placing the reference at the origin of this space {0,0,0}, one can visualise the effect of each process a datapoint corresponding to their effect in K,S and I.

**Fig. 5:**
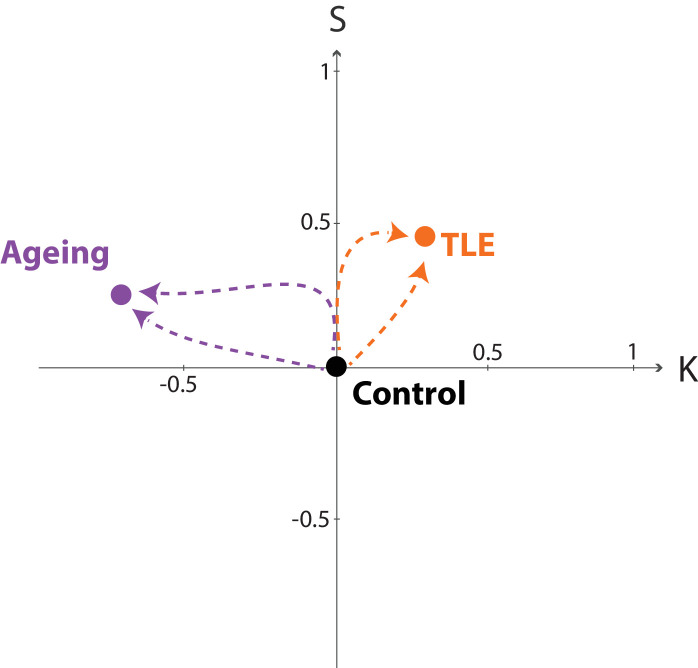
**Trajectories of morphology changes in health and disease.** Visualising the changes in ageing and TLE (same data as Fig. 4) in the 2D projection into K and S as trajectories from the origin. We chose to show a 2D projection of K×S×I space for simplicity. Both ageing and TLE process have been centered according to their respective control group. The respective datapoints are derived from the corresponding d values in each component from Fig. 4. Dashed lines indicate possible (hypothesised) trajectories. Note that trajectories can in theory move in any direction in this space, as the axes are now independent. Shared trajectories would reflect true shared mechanisms of brain morphology change.

Fig. 5 shows the control/reference as a point at the origin. Ageing and TLE are represented as two separate datapoints in this space, and clearly separated by the K component. In such a representation it becomes clear that both processes/conditions must have followed a trajectory (indicated by dashed lines in Fig. 5) that links the control condition with the disease or ageing “end points”. These trajectories could in theory follow any path, and are not restricted to particular parts of the space, as the variables are independent. The conceptual advance of this paper is to construct such a space where the axes are independent. This now allows for an unbiased study of disease trajectories (Jensen et al., 2014) on an individual, or group level. Clustering of trajectories now will reflect shared disease mechanisms, rather than unaccounted covariance between variables.

## 4 Discussion

With TLE and ageing as examples, we show limitations of using and interpreting morphological measures in a univariate manner. To account for the existing covariance between morphological variables, we suggest using new independent variables/measures. These independent measures clearly demonstrate that although TLE appears morphologically similar to the ageing process, these two processes are in fact distinct in terms of their morphological alterations. Thus, our example demonstrates that simple univariate analyses are unable to disambiguate the two processes, while our proposed change of coordinates could distinguish them.

Although we used a whole-hemisphere approach in our main figures, parallel arguments also hold for region-specific changes. We used the whole-hemisphere analyses to demonstrate the principle that covarying morphological measures need to be accounted for; however, we acknowledge that ageing and disease processes should not necessarily be simplified to a whole-hemisphere view when trying to understand their biological mechanisms. Indeed, we previously showed that the scaling law also holds for lobes/areas of the same brain (Wang et al., 2019b). This means that local measures of K,S and I can be derived, based on local measures of cortical thickness, total and exposed surface area. In Suppl. Text S3, we show that K, I, and S generally vary in a similar manner in different lobes as the whole hemisphere. We further note that, for example, the increase in K for TLE is most strongly seen in the temporal lobe. Thus, we demonstrate that our principle of using independent components of brain morphology, such as K, I, and S, still holds for regional analyses. To ensure that future work can apply our principle to different regions of the brain, we also made our MATLAB code available (https://github.com/cnnp-lab/CorticalFoldingAnalysisTools), including the processing of regionalised measures.

As an alternative to parcellating the brain into discrete regions, it is in theory also possible to derive a point-wise/voxel-wise estimate of K,S and I on the cortical surface. In the first instance, similar principles as the derivation of the local gyrification index (Schaer et al., 2008) could be followed. Furthermore, methods accounting for the spatial scale and size of the brain, such as suggested in Rabiei et al. (2017) may be additionally beneficial. Such point-wise estimates may help in the discovery of covert local abnormalities in future.

TLE and ageing differ most in the tension term K in our analysis. While ageing is associated with a decrease in K (in agreement with previous work (Wang, Necus, Kaiser, Mota, 2016, Wang, Necus, Rodriguez, Taylor, Mota, 2019)), TLE is associated with an increase in K compared to controls. In the theoretical derivation of the scaling law, K appears as a term proportional to stresses acting perpendicularly on the cortical grey matter surface (Mota and Herculano-Houzel, 2015). We have previously speculated this term to represent a pressure applied to the outside of the cortical tissue, and tension pulling from inside of the cortex (Bayly, Taber, Kroenke, 2014, Essen, 1997, Franze, 2013, Mota, Herculano-Houzel, 2015, Wang, Necus, Kaiser, Mota, 2016, Xu, Knutsen, Dikranian, Kroenke, Bayly, Taber, 2010). These speculations are currently untested and future work is required. We conclude here that there are a range of pathological processes at work in different brain conditions. Our proposed methods may help elucidate these processes in future, and provide biological and biophysical context for data-driven observations.

In the scaling law, we consider the variables of cortical thickness, cortical surface area, and exposed surface area. Other morphological variables such as cortical volume or intracranial volume are additional, frequently-used quantities. In particular, intracranial volume is often used as a covariate to account for “brain size”. However, these volume variables may well hold additional information not captured in the scaling law (see e.g. Wierenga et al., 2014). Additional metrics, such as curvature measures, are also often used to quantify brain morphology. To demonstrate that our principle also generalises to such a wider set of morphological variables, we performed a data-driven Principal Component Analysis in Suppl. Text S2. We could demonstrate, similar to K, I and S, that TLE and ageing display similar effects in some components, but differ substantially in other components. At present, it is less clear how those components can be interpreted, unlike K, I and S. We expect future studies, combining data-driven and mechanistic approaches to shed more light on the interpretation of these directions/components in brain morphology.

Our work has some conceptual parallels with and distinctions from a few established neuroimaging analysis approaches. One highly related approach is the study of brain allometry. Typically, brain allometry investigates how a morphological variable changes with the size of the brain (de Jong et al., 2016). Brain size is often captured by total brain volume, or intracranial volume, or hemispheric volume. For example, it has been shown that cortical surface area scales allometrically with brain size, as opposed to isometrically (Toro et al., 2008). Accounting for allometry also revealed in an example that localised morphological changes (in univariate analysis) could indeed be underpinned by a global effect (Toro et al., 2009). Allometric scaling has also been described in cortical folding metrics in the past in an empirical manner (Germanaud, Lefvre, Fischer, Bintner, Curie, des Portes, Eliez, Elmaleh-Bergs, Lamblin, Passemard, Operto, Schaer, Verloes, Toro, Mangin, Hertz-Pannier, 2014, Germanaud, Lefvre, Toro, Fischer, Dubois, Hertz-Pannier, Mangin, 2012). These empirically described allometric scaling laws account for existing covariance of the data, and represent an important step forward in the understanding of brain morphology. However, there is an important conceptual distinction to our theoretically derived scaling law. The allometric scaling laws describe associations with growth or size of the brain specifically, whereas the universal scaling law, more generally, describes associations between three morphological variables not exclusively or specifically measuring brain size. The scaling law further embodies a hypothesised mechanism of how the shape of the brain arises as a result of physical forces. Our work here has highlighted the importance of considering multiple covarying morphological variables in general, rather than only considering brain size as the independent variable.

Apart from covariance of morphological quantities, our work is also related to measures of “fractal dimension” of the brain shape (see e.g. Madan and Kensinger, 2016). Indeed, a natural way in which such a universal scaling law could arise would be if cortices were self-similar (in a statistical sense) down to some fundamental length scale proportional to cortical thickness, approximating a fractal with fractal dimension 5/2 (see Wang, Necus, Kaiser, Mota, 2016, Wang, Necus, Rodriguez, Taylor, Mota, 2019). This is in the same range as recent reports of the empirically measured fractal dimension (Madan, Kensinger, 2016, Madan, Kensinger, 2017). However, this is just an indication, not proof, of the hypothesized self-similarity. Future studies will have to demonstrate that the brain actually approximates a fractal object, by e.g. relating the scaling of a single cortex undergoing a process of iterated coarse-graining versus the scaling of different cortices. If confirmed, then additional concepts from fractal geometry could further enhance our analysis and understanding of the brain’s folded shape.

Another prominent approach that is also concerned with the covariance of morphological quantities is the so-called “structural covariance analysis” (Alexander-Bloch et al., 2013). In that approach, the covariance is measured between different regions of the brain in terms of one morphological measure (e.g. cortical thickness), essentially assessing which regions change together across subjects. The popular approach is to then understand the covariance as a matrix that describes a network, and compare these networks between groups. These co-relationships between brain regions can also be non-linear, and manifold learning techniques have been applied to elucidate them (see e.g. Valk et al., 2020). The independent variables K, S and I may be more advantageous in terms of its reliability and comparability (Carmon et al., 2019) for structural covariance analysis.

A related approach has been termed “morphometric similarity”, in which for a single subject, the covariance between brain region is derived based on their similarity across a range of morphological measures (Seidlitz et al., 2018). Again, non-linear variants of this approach have also been formulated (for example on the similarity of morphometric distance matrices Soussia and Rekik, 2018). However, note that both types of approaches are concerned with covariance between brain regions, rather than covariance between morphological measures. We envisage that a comprehensive framework for cortical morphology would encompass both aspects in the future, and manifold learning approaches (see e.g. Aljabar, Wolz, Srinivasan, Counsell, Rutherford, Edwards, Hajnal, Rueckert, 2011, Gerber, Tasdizen, Joshi, Whitaker, 2009) may aid efforts in terms of data-driven discoveries.

Finally, we proposed the notion of “trajectories” in morphological space (spanned by independent variables), building on related previous work (e.g. Young et al., 2018). A key implication of such trajectories is that different brain processes (or disorders) may cluster in terms of their trajectories, or share parts of their trajectories, potentially indicating shared drivers/pathways/modulations (Jensen, Moseley, Oprea, Ellesøe, Eriksson, Schmock, Jensen, Jensen, Brunak, 2014, Taylor, Moreira da Silva, Blamire, Wang, Forsyth, 2020). Especially with a comprehensive region-specific and cross-region analysis of cortical morphology we expect clusters of directions to emerge. On an individual subject level, our approach may also help to develop more sensitive and specific biomarkers. Moreover, current efforts to relate morphological alterations to genetic alterations (e.g. Seidlitz et al., 2019) may help to develop an atlas of principal trajectories, and shed light on potential corresponding biological mechanisms.

In summary, our work represents a significant conceptual advance by contributing independent cortical morphology measures that can be interpreted without being hampered by other unaccounted morphological covariates. Using these independent measures we demonstrated that temporal lobe epilepsy, which appeared to resemble premature ageing in terms of cortical morphology, is in fact characterised by distinct morphological changes from ageing. The same principle may resolve some of the existing confusion in the literature regarding morphology alteration in other brain conditions and processes. In future, we hope that systematic studies of brain morphology can be associated with the underpinning biological mechanisms, applied on a regional basis in cross-sectional and longitudinal studies to become a useful tool in biomarker development and understanding the brain in health and disease.

## Author contributions

YW, PNT, and BM conceived the idea. YW wrote the code, performed all the analysis, and produced all the figures. TL validated the code. GW, SBV, JdT, and JSD contributed the TLE data. PNT processed the TLE data. YW, TL, BL, JHN, and PNT inspected the Freesurfer processing of the data and performed manual corrections where needed. YW, PNT, and BM drafted the manuscript. All authors participated in critically reviewing and revising the manuscript.

## Code and data availability

Code for extraction of raw cortical measures can be found on Zenodo: https://zenodo.org/record/3608675#.XjA80-f7TUI and Github: https://github.com/cnnp-lab/CorticalFoldingAnalysisTools. Data underlying the figures in this paper and the corresponding code can be found on Github: https://github.com/cnnp-lab/2020Wang_TLEFoldingHemi

## CRediT authorship contribution statement

**Yujiang Wang:** Conceptualization, Methodology, Software, Validation, Formal analysis, Investigation, Resources, Data curation, Writing - original draft, Writing - review & editing, Visualization, Project administration. **Karoline Leiberg:** Validation, Investigation, Formal analysis, Visualization, Writing - review & editing. **Tobias Ludwig:** Software, Validation, Data curation, Writing - review & editing. **Bethany Little:** Data curation. **Joe H Necus:** Validation, Data curation. **Gavin Winston:** Resources, Writing - review & editing. **Sjoerd B Vos:** Resources, Writing - review & editing. **Jane de Tisi:** Resources, Data curation, Writing - review & editing. **John S Duncan:** Resources, Writing - review & editing. **Peter N Taylor:** Conceptualization, Methodology, Software, Validation, Resources, Data curation, Writing - review & editing. **Bruno Mota:** Conceptualization, Methodology, Writing - review & editing.

## Appendix A Supplementary materials

Supplementary Data S1 Supplementary Raw Research Data. This is open data under the CC BY license http://creativecommons.org/licenses/by/4.0/ Supplementary Data S1
