# Supplementary material for: Independent components of human brain morphology
Source: Neuroimage. 2021 Feb 1;226:117546. doi: 10.1016/j.neuroimage.2020.117546 (PMC7836233; doi:10.1016/j.neuroimage.2020.117546)
Supplement: Supplementary Data S1 — Supplementary Raw Research Data. This is open data under the CC BY license http://creativecommons.org/licenses/by/4.0/ [file mmc1.pdf]

Supplementary Text S1: Principal Component Analysis for healthy ageing and TLE

A second set of independent measures, besides K, I and S, for the comparison of the two processes TLE and ageing can be provided by a principal component analysis. We derive the principal components from the reference cohorts of the two data sets and look at the changes in the principal component space due to the effects of ageing and TLE.

The data is preprocessed in the same way as for the analysis in KIS space, taking logarithms of the variables A_t, A_e and T, and regressing out sex from the ageing data, and sex and age from the TLE data. We also transform the variable $\log T$ to $\log T^2$ to only use measures of area and allow for better comparison with the KIS analysis. The data is standardised as z-scores with respect to the mean and standard deviation of its respective reference cohort.

The PCA is performed on the reference cohorts of both sets individually. For the ageing cohort, the loading vectors are:

$$
\begin{aligned}
l_{age,1} &= 0.71 \log A_t + 0.70 \log A_e – 0.10 \log T^2 \\
l_{age,2} &= -0.01 \log A_t + 0.14 \log A_e + 0.99 \log T^2 \\
l_{age,3} &= 0.71 \log A_t - 0.70 \log A_e + 0.11 \log T^2
\end{aligned}
$$

For the TLE cohort, the loading vectors are:

$$
\begin{aligned}
l_{TLE,1} &= 0.71 \log A_t + 0.70 \log A_e – 0.09 \log T^2 \\
l_{TLE,2} &= -0.04 \log A_t + 0.16 \log A_e + 0.99 \log T^2 \\
l_{TLE,3} &= 0.70 \log A_t - 0.70 \log A_e + 0.14 \log T^2
\end{aligned}
$$

There are no major differences in the individual loading matrices. This justifies a PCA based on the combined reference cohorts, which is used for the further analysis. The loading vectors are then:

$$
\begin{aligned}
l_{TLE,1} &= 0.71 \log A_t + 0.70 \log A_e – 0.09 \log T^2 \\
l_{TLE,2} &= -0.02 \log A_t + 0.15 \log A_e + 0.99 \log T^2 \\
l_{TLE,3} &= 0.71 \log A_t - 0.70 \log A_e + 0.12 \log T^2
\end{aligned}
$$

The first PC seems to be a weighted average of the two surface area measures $\log A_t$ and $\log A_e$, and could therefore be interpreted as the size of the surface area. The second loading vector is dominated by the cortical thickness $\log T^2$, and also includes the exposed surface area. Subjects have a high PC2 score if their cortical surface is thick and large in terms of exposed area. The third PC has $\log A_e$ as the only variable with a negative coefficient, and thus appears to be the contrast between exposed surface area and the two other measures, total surface area and thickness. High scoring subjects will have a thick cortical surface with a high gyrification index.

The first two loading vectors are rotated compared to I and S. Since those variables were chosen for interpretation, we would not necessarily expect them to arise from a PCA. The third loading vector is the most similar to K, albeit with a slight angle between the vectors of about 30 degrees. Importantly, we tend to observe a coefficient around 0.17 for the cortical thickness (relative to a coefficient of 1 for $\log A_t$), which also fluctuates slightly between datasets. The theoretically derived coefficient for cortical thickness is 0.25, and with this assumption, we could show in Fig 2C in the main manuscript that the coefficient for $\log A_e$ is then derived to be 1.25 from the data. Some discrepancy due to noisy measurements is expected between the PCA derived loading vectors and the scaling law. Further, we speculate that the reasons for the slight, and possibly systematic deviation from the scaling law is due to the simplifying assumptions made in the scaling law (e.g. homogeneous spatial distribution of thickness and surface area).

Nevertheless, the important question for biological and clinical applications is that the new coordinates are sensitive to biological effects and provide meaningful insight. To test this, we transformed both ageing and TLE dataset into the PC space. We can examine the changes associated with TLE and ageing in the distributions of the bootstrapped means of the z-scores (Figure S1). In both processes, the scores in the first PC are not substantially altered. The scores in PC2 decrease in both processes, with $d = -0.74$ for TLE and $d = -0.69$ for ageing. Only in the third PC, we observed opposing changes: For ageing, the scores in PC3 decrease ($d = -0.15$), whereas they increase for TLE ($d = 0.15$). Note that these effect sizes in PC3 are substantially smaller than in K, indicating that K is a more sensitive measure for the differing biological effects in TLE and ageing.

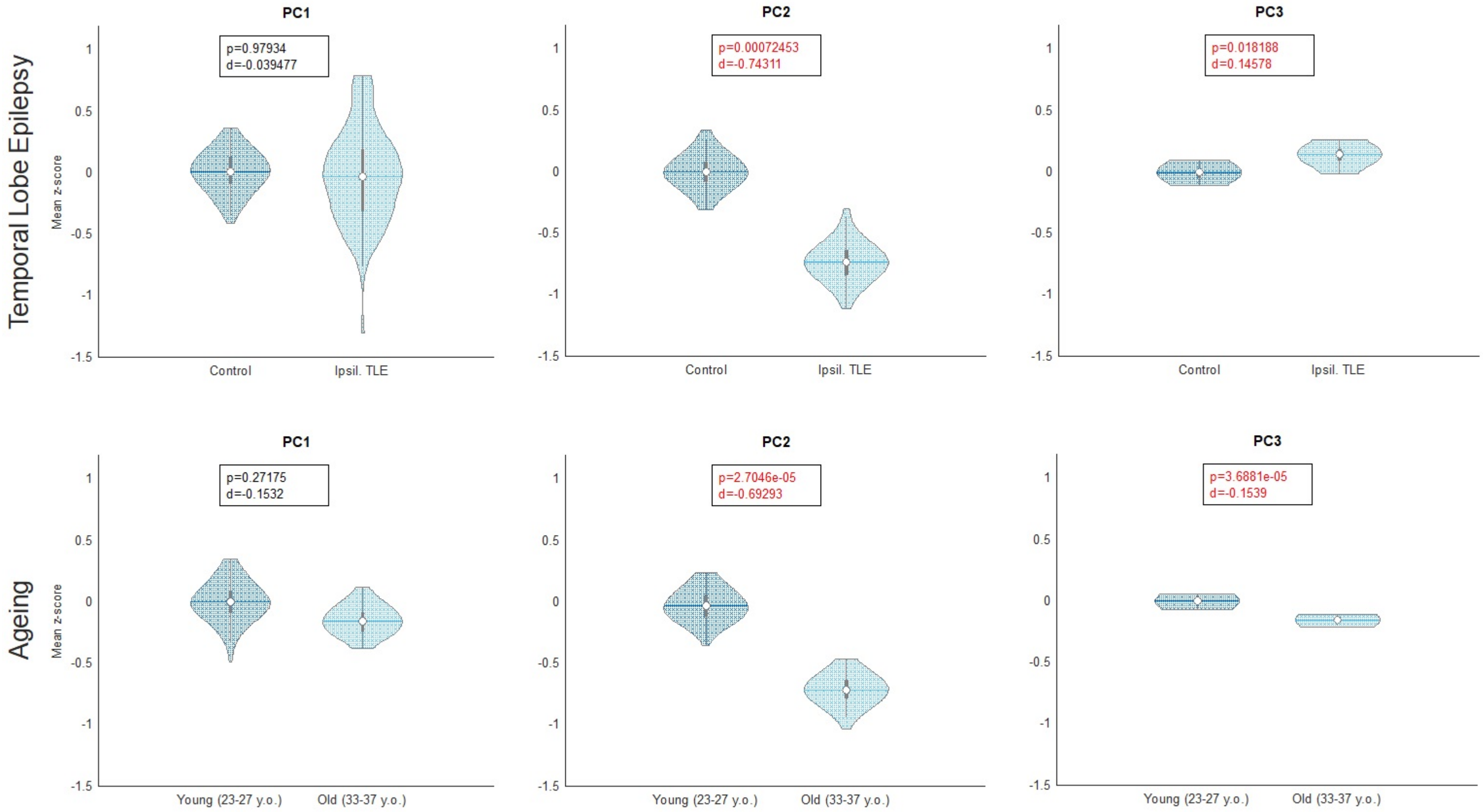

Figure S1: Morphological changes in the principal components for TLE compared to healthy ageing.

Supplementary Text S2: PC Analysis for healthy ageing and TLE including more components

To demonstrate the generalisability of our results to a wider set of morphological measures, we performed a principal components analysis including the measures grey matter volume V_G, white matter volume V_W and mean curvature MC additionally to the total surface area A_t, exposed surface area A_e and cortical thickness T, to assess whether the analysis holds up in a multivariate space of higher dimensions. Note that of course more morphological measures could be added (given sufficient number of observations). Here, we used 6 different measures as an illustrative example.

The grey matter volume is calculated as the volume encapsulated within the pial and white matter surface mesh from FreeSurfer. The white matter volume is calculated as the volume encapsulated within the white matter surface mesh from FreeSurfer. Note that this also includes subcortical structures and ventricles, and is perhaps best understood as the volume inside the grey matter ribbon. The mean curvature of each hemisphere is computed as a weighted average of the mean curvature of its lobes, with the weights being supplied by the surface area of the lobes.

As before, we take logarithms of the variables, and regress out the covariates sex from the ageing data set, and both sex and age from the TLE data. We convert $\log(T)$ to $\log(T^2)$ and standardise the data as z-scores relative to the reference cohorts.

Performing a PCA on the ageing cohort, we obtain the loading vectors

$$
\begin{aligned}
l_{age,1} &= 0.50\log(A_t) + 0.51\log(A_e) - 0.01\log(T^2) + 0.50\log(V_G) + 0.48\log(V_W) - 0.05\log(MC)\\
l_{age,2} &= -0.09\log(A_t) + 0.01\log(A_e) + 0.71\log(T^2) + 0.15\log(V_G) - 0.13\log(V_W) - 0.67\log(MC)\\
l_{age,3} &= -0.03\log(A_t) + 0.05\log(A_e) + 0.64\log(T^2) + 0.16\log(V_G) - 0.09\log(V_W) + 0.74\log(MC)\\
l_{age,4} &= -0.52\log(A_t) + 0.12\log(A_e) + 0.15\log(T^2) - 0.33\log(V_G) + 0.76\log(V_W) + 0.01\log(MC)\\
l_{age,5} &= -0.26\log(A_t) + 0.85\log(A_e) - 0.07\log(T^2) - 0.23\log(V_G) - 0.39\log(V_W) - 0.01\log(MC)\\
l_{age,6} &= -0.63\log(A_t) - 0.05\log(A_e) - 0.23\log(T^2) + 0.73\log(V_G) - 0.06\log(V_W) + 0.01\log(MC)
\end{aligned}
$$

The loading vector derived from the TLE cohort are

$$
\begin{aligned}
l_{TLE,1} &= 0.50\log(A_t) + 0.52\log(A_e) + 0.01\log(T^2) + 0.50\log(V_G) + 0.48\log(V_W) + 0.02\log(MC)\\
l_{TLE,2} &= -0.17\log(A_t) + 0.04\log(A_e) + 0.70\log(T^2) + 0.13\log(V_G) + 0.01\log(V_W) - 0.68\log(MC)\\
l_{TLE,3} &= 0.12\log(A_t) - 0.13\log(A_e) + 0.59\log(T^2) + 0.32\log(V_G) - 0.37\log(V_W) + 0.62\log(MC)\\
l_{TLE,4} &= -0.52\log(A_t) + 0.25\log(A_e) + 0.29\log(T^2) - 0.32\log(V_G) + 0.58\log(V_W) + 0.39\log(MC)\\
l_{TLE,5} &= -0.11\log(A_t) + 0.81\log(A_e) - 0.03\log(T^2) - 0.20\log(V_G) - 0.54\log(V_W) + 0.00\log(MC)\\
l_{TLE,6} &= -0.65\log(A_t) + 0.03\log(A_e) - 0.29\log(T^2) + 0.70\log(V_G) - 0.07\log(V_W) + 0.00\log(MC)
\end{aligned}
$$

The loading matrices are relatively similar, especially in the first two, and

last loading vectors. We thus do a PCA on the combined reference cohorts. The loading vectors are then:

$$l_{TLE,1} = 0.50\log(A_t) + 0.52\log(A_e) + 0.00\log(T^2) + 0.50\log(V_G) + 0.48\log(V_W) - 0.02\log(MC)$$
$$l_{TLE,2} = -0.12\log(A_t) + 0.02\log(A_e) + 0.71\log(T^2) + 0.15\log(V_G) - 0.07\log(V_W) - 0.67\log(MC)$$
$$l_{TLE,3} = 0.04\log(A_t) - 0.03\log(A_e) + 0.61\log(T^2) + 0.24\log(V_G) - 0.23\log(V_W) + 0.72\log(MC)$$
$$l_{TLE,4} = -0.53\log(A_t) + 0.21\log(A_e) + 0.22\log(T^2) - 0.35\log(V_G) + 0.69\log(V_W) + 0.19\log(MC)$$
$$l_{TLE,5} = -0.14\log(A_t) + 0.83\log(A_e) - 0.04\log(T^2) - 0.25\log(V_G) - 0.48\log(V_W) + 0.01\log(MC)$$
$$l_{TLE,6} = -0.66\log(A_t) + 0.04\log(A_e) - 0.26\log(T^2) + 0.70\log(V_G) - 0.08\log(V_W) + 0.01\log(MC)$$

With a couple of exceptions, the directions do not offer any straightforward interpretations. The first PC, however, is a weighted average of the two surface area measure $\log(A_t)$ and $\log(A_e)$ and the two volume measures $\log(V_G)$ and $\log(V_W)$. It can be thought of as a measure of the overall size of the hemisphere. The second PC is a contrast between the cortical thickness and the curvature. Subjects will have a high score in this PC if the cortical surface is thick and relatively flat.

The first three PCs combined explain 96% of the variation in the data.

We transform the data into PC space and plot the distribution of its bootstrapped means (Figure 1). The two processes TLE and ageing behave very similarly in PC1, PC2, PC5 and PC6. Ageing decreases scores in the third PC significantly ($d = -0.55$), whilst there is no change caused by TLE ($d = 0.02$). In PC4, ageing increases the scores with $d = 0.18$, however they decrease for TLE with $d = -0.40$.

This shows that our results in the three-dimensional space of K, I and S also transfers to higher dimensions: The processes ageing and TLE behave similarly in some PCs. Especially PC1, which is perhaps most similar to I, shows similar effect sizes to I in both TLE and ageing. However, the two processes also have clear distinctions in their effect on other linearly independent PCs. At present, it is less clear how those PCs can be interpreted in this analysis, unlike K. We expect future studies, combining data-driven and mechanistic approaches to shed more light on the interpretation of these directions/components in brain morphology.

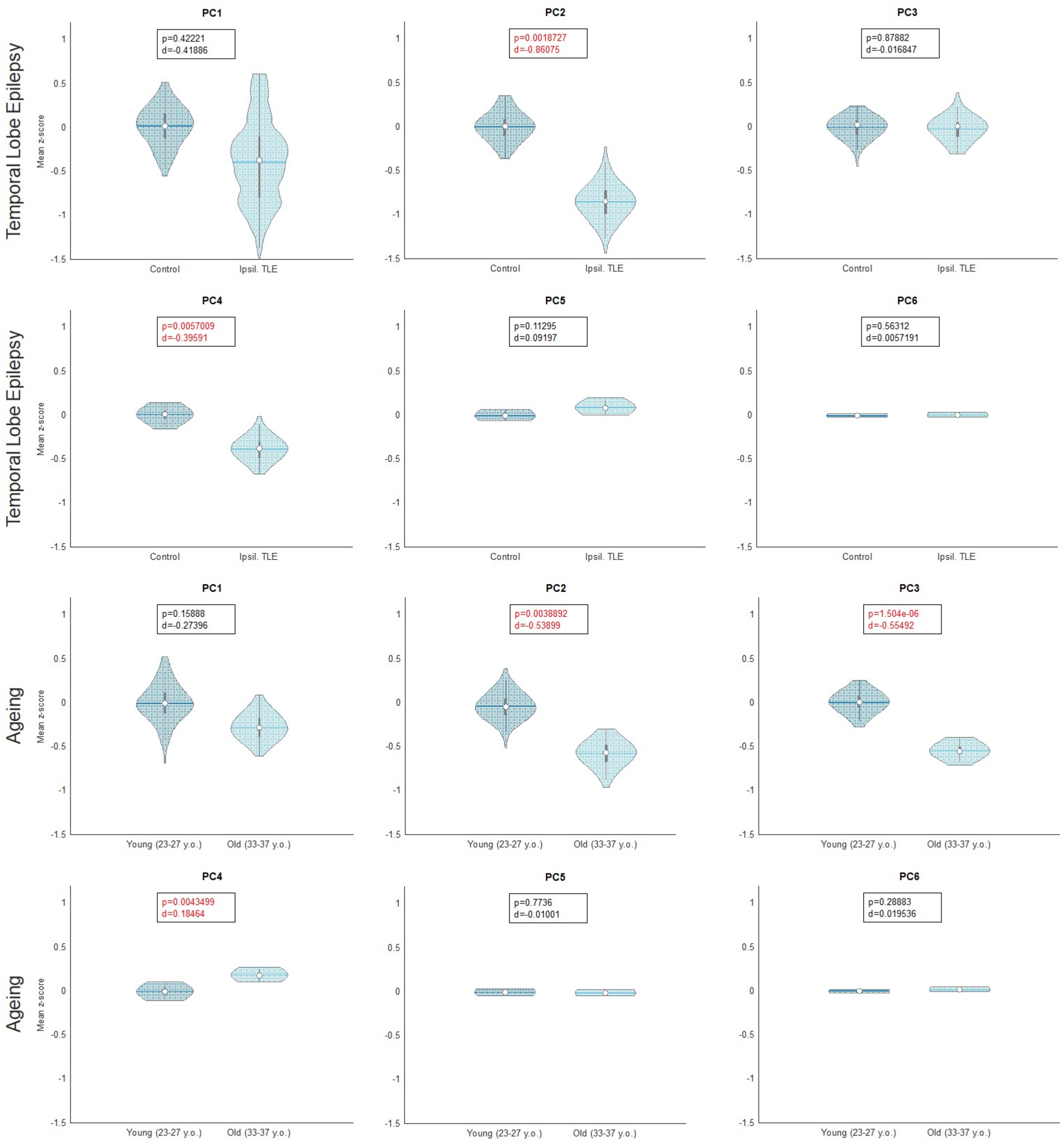

Figure S2: **Morphological changes in the principal components for TLE compared to healthy ageing.**

Supplementary Text S3: Independent components for the morphology of cortical lobes in healthy ageing and TLE

To extend our analysis on entire hemispheres, we also show an example here of how the same principles apply to local, regional morphological measures. For this, we use the partition of the hemisphere into frontal, temporal, parietal and occipital lobe. We also used the ipsilateral hemisphere only in TLE patients, to be consistent with our main figures.

Due to the issue of partition sizes affecting the comparability of lobes and their abidance by the scaling law as described in Wang *et al.* 2019, we rescale the total surface area A_t and the exposed surface area A_e for each lobe to what their values of a whole hemisphere would be, using the Gaussian curvature as the indicator of proportion to reconstruct the hemisphere:

$$A'_t = A_t \frac{4\pi}{I_G},$$

where A'_t is the reconstructed total surface area of the lobe, A_t is the observed total surface area of the lobe, and I_G is the integrated Gaussian curvature of the lobe. Similarly, a correction is also applied to A_e.

We take logarithms of the corrected variables and regress out sex from the ageing data, and sex and age from the TLE data. We then convert to z-scores with respect to the respective reference cohort and compute their bootstrapped means. The difference between the mean of each comparison cohort and that of its respective reference cohort is shown in the figures S3 - S5.

The effects of TLE and ageing in the frontal lobe and the temporal lobe are very similar to what was observed for the whole hemisphere: With both processes I decreases and S increases, and in K they have opposing effects with TLE causing an increase and ageing causing a decrease.

In the parietal lobe, both processes show the same alterations in S as above, but the changes in I and K are different. Both variables decrease with ageing, as was the case in the frontal lobe, the parietal lobe, and the hemisphere, but in the parietal lobe TLE leaves I and K unchanged.

In the occipital lobe, TLE was associated with an increases in K and decreases in S and I. The effect of ageing is again opposite, with decreases in K, increases in S and I unchanged.

We expected TLE to most strongly affect the temporal lobe, which is indeed the case for K. However in terms of size, I, the occipital lobe is most affected in TLE, and the shape S appears most altered in the parietal lobe in TLE. It is conceivable that these strong changes in I and S in other lobes arises as a result of mechanical changes (K) in the temporal lobe, and future longitudinal studies are required. Furthermore, it is also well-known that TLE patients show widespread changes in their brain structure and morphology beyond the temporal lobe.

In ageing, we note the strongest effect in K in the frontal lobe, whereas the effects in I and S are more evenly distributed across the lobes. The smallest effects of ageing in K, I, and S are always in the occipital lobe.

Thus, the effects of ageing and TLE differ in different lobes. The difference between the processes across all lobes is mainly due to differences in K.

It is theoretically possible to use a finer-grained parcellation of the brain than the lobes. However, in our previous work we noted that information about the local geometry of the cortex is also required, and a fine-grained parcellation that is smaller than a gyrus is therefore not recommended. To make this extension of our work accessible to others, we have also made our MATLAB code available (`https://github.com/cnnp-lab/CorticalFoldingAnalysisTools`), including the processing of regionalised measures.

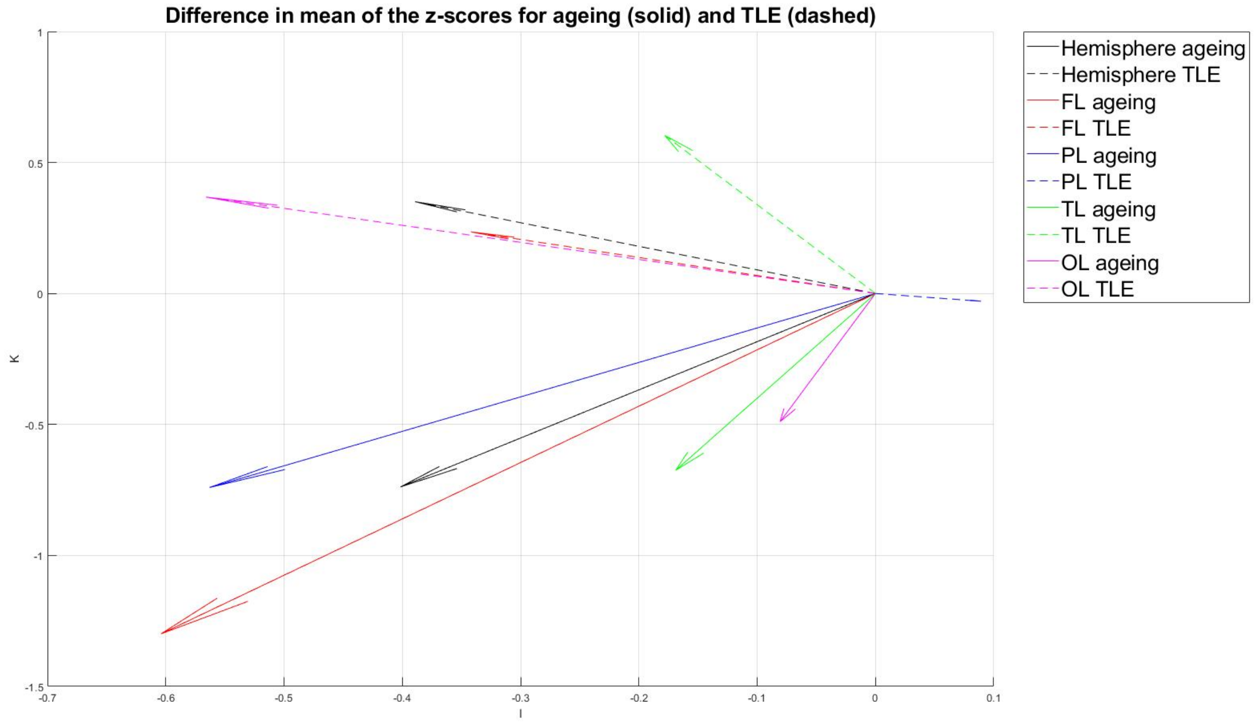

Figure S3: **Difference between means of comparison cohorts and reference cohorts in I and S. The solid lines represent the ageing process, whilst the dashed lines represent TLE.**

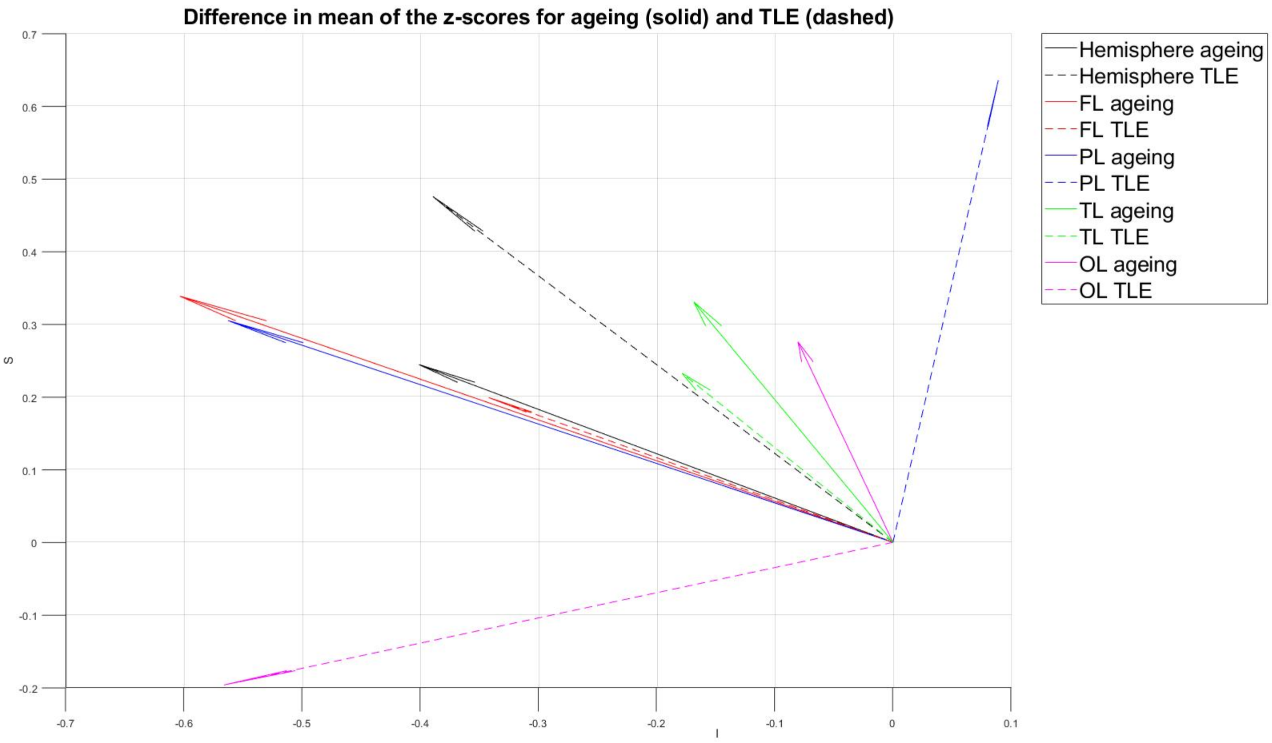

Figure S4: **Difference between means of comparison cohorts and reference cohorts in I and K.**

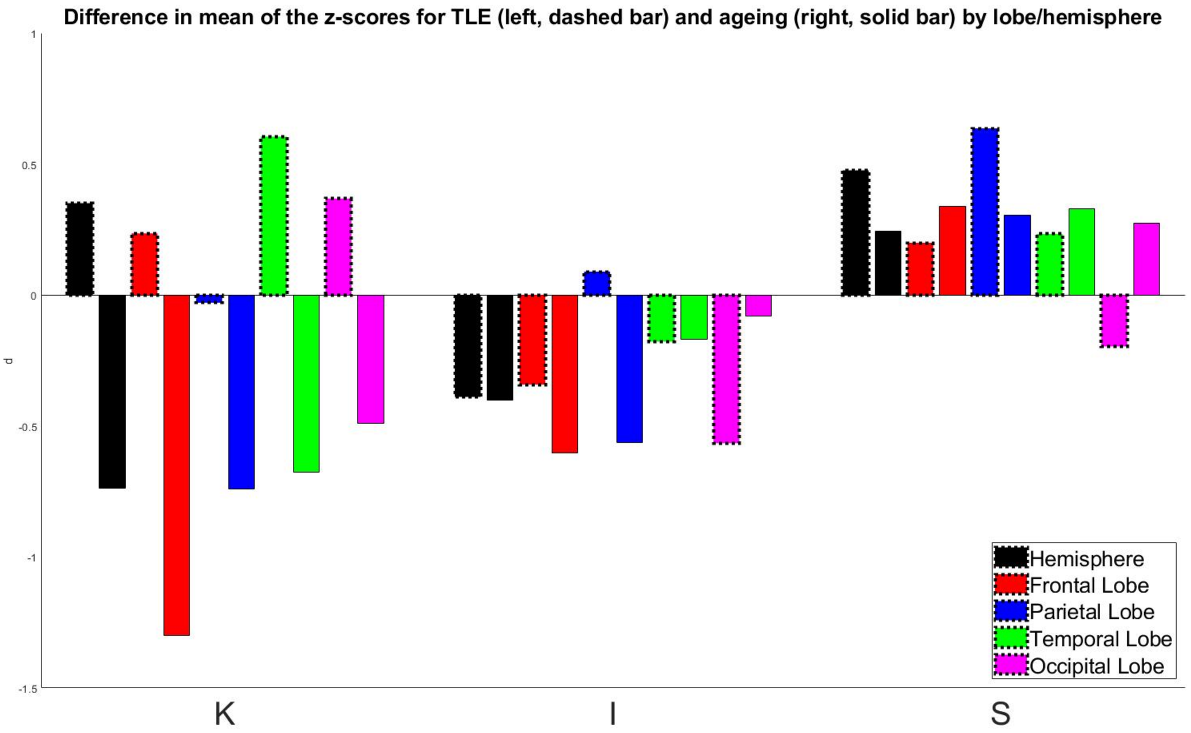

Figure S5: **Comparison of the effect of TLE (dashed bars, left) vs ageing (solid bars).**
